# Solitary peripheral ivory osteoma of the mandible presenting with difficulty in deglutition: a case report

**DOI:** 10.15171/joddd.2017.011

**Published:** 2017-03-15

**Authors:** Kumar Nilesh, Aaditee V. Vande, Suresh K. Veerabhadrappa

**Affiliations:** ^1^Department of Oral and Maxillofacial Surgery, School of Dental Sciences, KIMSDU, Karad, India; ^2^Department of Prosthodontics, School of Dental Sciences, KIMSDU, Karad, Maharashtra, India; ^3^Faculty of Dentistry, SEGi University, Selangor, Malaysia

**Keywords:** Jaw, osteogenic tumor, radiopaque lesion, swallowing

## Abstract

Osteomas are benign bone tumors which arise from the cortex or medulla of craniofacial and jaw bones. They are usually asymptomatic or present as slow-growing painless masses. Larger lesions may present with aesthetic (facial asymmetry) and functional disturbances (jaw deviation, difficulty in breathing, pain, and sensory deficits). This paper highlights a case of solitary peripheral osteoma composed of a compact bony mass arising from the lower border of the mandible in an adult female patient. The lesion presented with discomfort during deglutition, which was attributed to impingement of muscles of the oral cavity floor, including the anterior belly of digastric muscle.

## Introduction

Osteomas are rare benign neoplasms composed of compact or cancellous bone. They commonly involve cranial and facial skeleton, including the paranasal sinuses and jaw bones. Based on their site of origin, osteomas are classified as peripheral (periosteal) and central (endosteal). Peripheral osteomas are more common and arise from the cortical bone, whereas central variants develop within the endosteum of bone. Rarely, osteomas can develop as extra-skeletal lesions located in soft tissues, commonly in muscles.^1^

Osteomas invariably present as a slow-growing single bony mass (solitary lesion) with a sessile or pedunculated base. Multiple osteomas of the jaw may be present as part of Gardener syndrome along with features like intestinal polyps, multiple supernumerary/impacted teeth and tumors of soft tissues.^2^ Osteomas might be composed of either dense compact bone with scanty marrow spaces (compact/ivory osteoma) or cancellous bone with abundant bony trabeculae along with fibro-fatty marrow tissue (cancellous/trabecular/spongy osteoma).

Peripheral osteomas (PO) may go unnoticed when small or present as painless slow-growing bony hard masses often projecting as mushroom-shaped outgrowths from the outer cortex of the jaw. Larger mandibular lesions may cause pain, facial asymmetry, deviation of the jaw or difficulty in breathing.^3,4^ This paper reports a case of PO in an adult female patient, arising from the lower border of the mandible. The bony mass presented with discomfort during swallowing due to impingement of the muscles of the oral cavity floor and the anterior belly of the digastrics muscle.

## Case report

**Figure 1.**
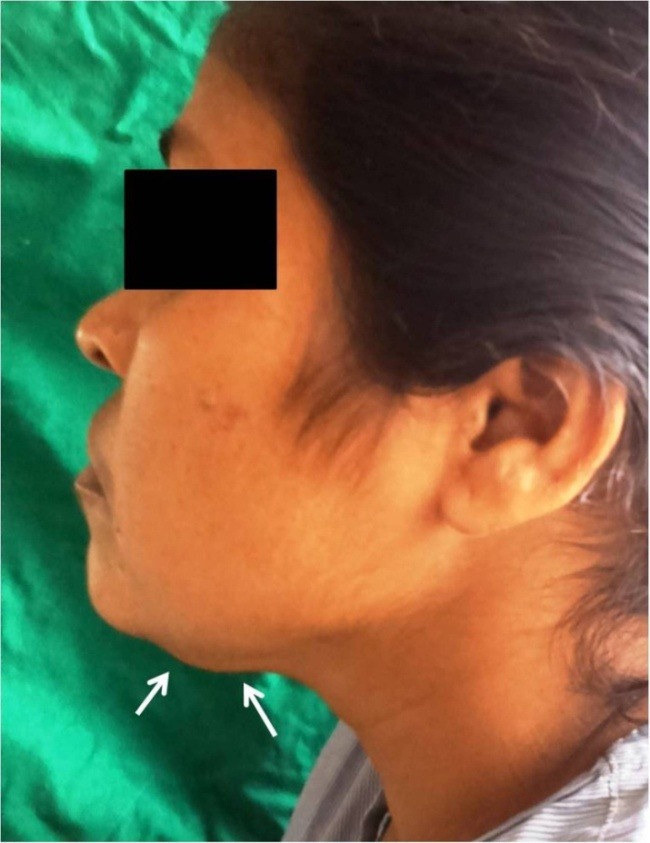
Clinical image showing a bony swelling over the lower border of the mandible.

A 40-year-old Indian female consulted our oral and maxillofacial clinic, complaining of discomfort during swallowing. The patient was apparently alright two months back when she developed mild discomfort during deglutition. Around the same time the patient also noticed a painless swelling over the upper left side of the neck, which gradually kept increasing in size. No significant medical history or history of previous trauma was reported. On extraoral examination, no gross facial asymmetry was noticed. Intraoral examination showed upper and lower jaws with normal teeth. No other mucosal abnormality was detected over oral and oropharyngeal mucosa. Palpation of the inferior border of the mandible revealed a painless bony hard mass over the inferior border of the mandible in relation to the left canine‒premolar region. The growth was fixed to the inferior border and lingual surface of the mandible and did not show any movement during deglutition (Figure 1). Cervical lymph nodes were not palpable.

**Figure 2.**
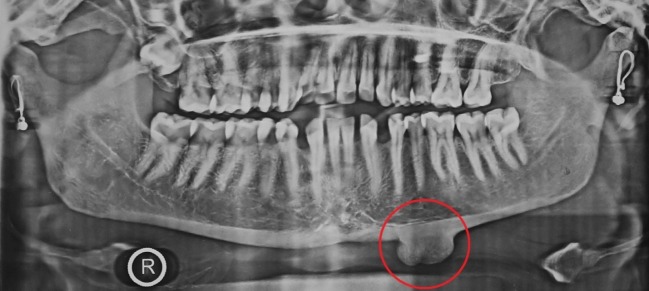
Panoramic radiographic view showing a radiopaque lesion (circle).

**Figure 3.**
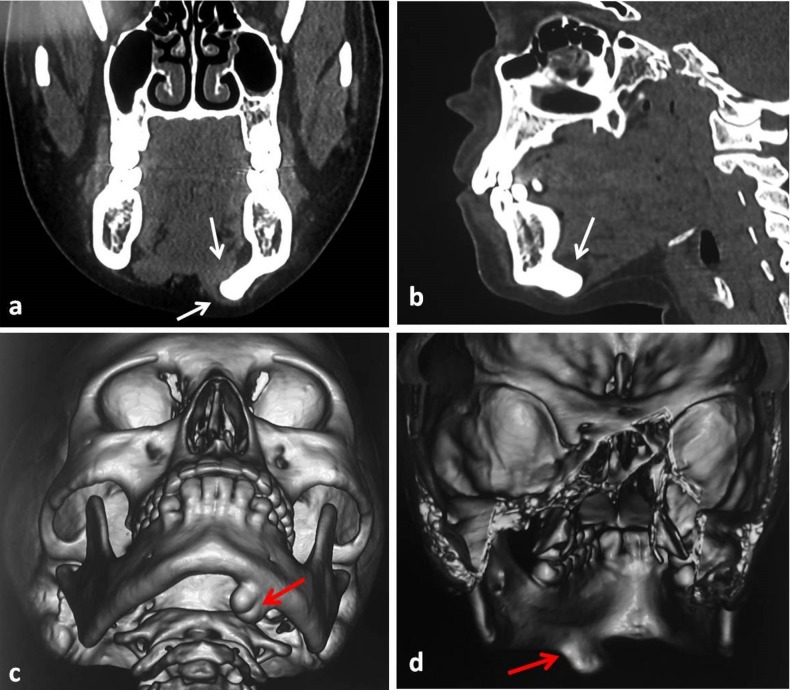
Computed tomogram showing a hyperdense growth impinging over the muscle plane in the oral cavity floor (a,b). 3D formatted images (c,d).

An orthopantomogram showed a well-defined homogenous radiopaque mass arising from basal part of the mandible below the apices of the left premolar and the first molar teeth with a continuous contour (Figure 2). Computed tomography (CT) scan with sectional and 3D formatted images was obtained for further evaluation of the lesion. CT scan showed a well-defined, hyperdense mass measuring 2.5×2 cm in size, arising from the left lower border of the mandible. Coronal and sagittal sections showed the growth projecting slightly towards the lingual aspect and impinging over the muscle plane on the oral cavity floor and neck (Figure 3). No other lesion was seen involving the craniofacial skeleton. Based on the clinical and radiological findings, provisional diagnosis of peripheral osteoma was made.

**Figure 4.**
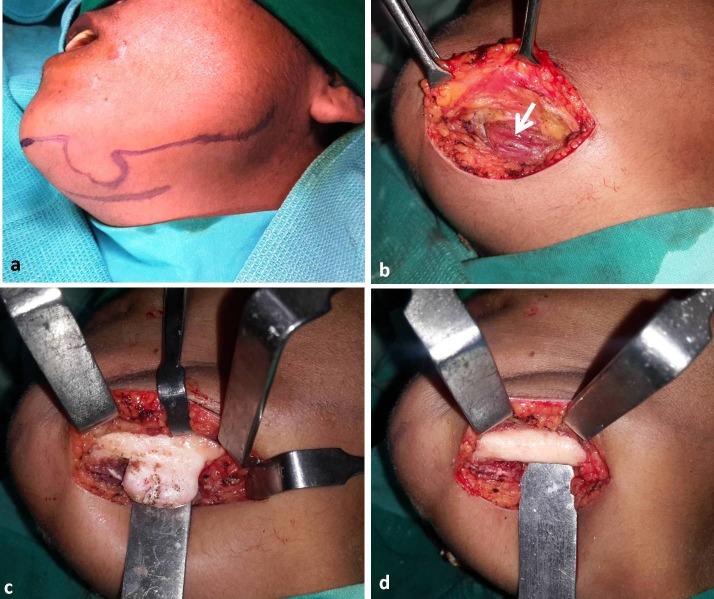
Surgical steps; incision (a), anterior belly of digastric muscle (arrow) overriding the lesion (b), surgical exposure (c), and excision of lesion (d).

**Figure 5.**
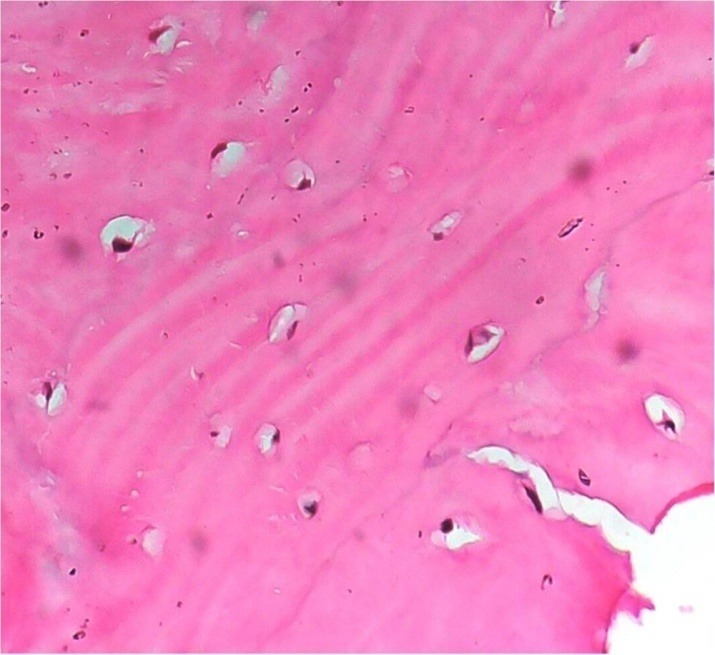
Photomicrograph showing dense compact bone with osteocytes (40 X magnification).

**Figure 6.**
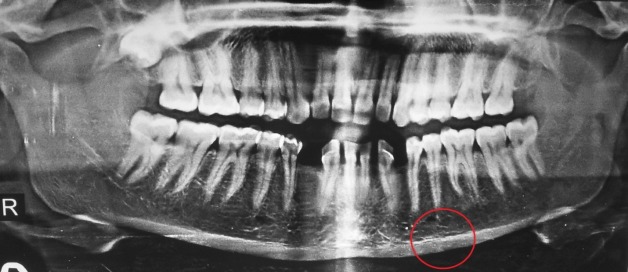
Panoramic radiograph, one year postoperative.

Surgical excision of the tumor was planned and executed under general anesthesia. The surgical approach used was submandibular incision placed in the upper neck crease. Upon layered dissection over the lower border of the mandible, the anterior belly of the digastric muscle was seen overriding the lesion on its buccal aspect (Figure 4). The muscle was retracted medially by blunt dissection. The lesion was isolated from surrounding muscles and excised. The excised specimen was submitted for histopathological analysis. Microscopic sections of the specimen showed compact bone having mature lamellae with scanty marrow (Figure 5). Based on clinical presentation, imaging characteristics and histological features a final diagnosis of solitary peripheral ivory osteoma of the mandible was reached. The patient was recalled at regular intervals and showed complete resolution of symptom. A follow-up panoramic view taken at one-year postoperative interval showed complete healing of the cortical aspect of the inferior border of the mandible with no sign of recurrence (Figure 6).

## Discussion

Osteomas are defined as benign osteogenic lesions predominantly composed of osteoblastic connective tissue which forms abundant osteoid and new bone that eventually mature to compact bone over a period of time.^5^ Based on its site of origin they can be classified as central (arising from the medullary cavity) or peripheral (arising from the cortex). Osteomas in the head and neck region frequently involve paranasal sinuses and frontal bone. Other bones involved include the temporal bone, orbit, external auditory canal and pterygoid process. PO of the jaw skeleton is more commonly seen in the mandible as compared to maxilla. Sayan et al in a review of 35 PO cases found that the majority of the lesions involved the frontal bone (28.57%), with the mandible and maxilla accounting for 22.85% and 14.28% of cases, respectively.^6^ Mandibular PO occurs predominantly in the body‒ramus region and condyle. Those arising from the dentate segment may involve the buccal/lingual cortex or the lower border of the mandible. In the present case the lesion developed from the basal bone of the mandible in the premolar molar region. Cases of PO arising from the coronoid process and sigmoid notch have also been reported in the literature.^7^

The pathogenic process of osteoma is not clearly understood. Some authors classify it as a true neoplasm while others consider it as a reactive lesion secondary to chronic irritation or trauma.^2^ Osteomas show a slow continuous increase in size without growth cessation at adulthood. This characteristic feature helps distinguish osteomas from other bony exostoses and supports a neoplastic origin. In the present case no history of trauma or any overt cause of chronic irritation could be identified, possibly suggesting neoplastic etiology.

Clinically, mandibular PO presents as an asymptomatic slow-growing bony hard mass which may be diagnosed during routine radiographic examinations. As the lesion grows in size, it causes an intraoral (vestibular/lingual) swelling or an extraoral mass causing facial asymmetry.^3,4^ Depending upon its location, mandibular PO may cause other functional disturbances like occlusal dysfunction, pain, sensory deficit and ulceration of the overlying mucosa. Condylar osteomas produce limited mouth opening and mandibular deviation. A case of large PO of the mandible causing facial asymmetry, deviation of the tongue and respiratory obstruction has been previously reported.^8^ None of the previous cases of PO of the mandible have been reported to present with difficulty swallowing as in this report. CT imaging showed the lesion impinging on the muscles of oral cavity floor. Surgical excision is the treatment of choice for PO. Recurrence is relatively infrequent and malignant transformation has not been reported.

## Conclusion

Presentation of PO depends upon the size and location of the pathology. The majority of the cases are asymptomatic or presents as a slow-growing bone mass. Larger lesions may cause intraoral or extraoral swelling with facial asymmetry. Other less frequently reported symptoms include difficulty in breathing, deviation of the tongue and ulceration of the superficial mucosa. This paper highlights a rare presentation of PO of the mandible causing difficulty during deglutition due to impingement of muscles of the oral cavity floor.

## Acknowledgments

The authors would like to thank Dr. Ajay Nayak, associate professor, Department of Oral Medicine and Radiology, School of Dental Sciences, Karad, India.

## Authors’ contributions

KN preformed the surgical procedure. KN and SV were involved in diagnosis and formatting the case report. AV drafted the manuscript and all the authors contributed to the revision and final approval of the manuscript.

## Funding

The authors report no funding for this article.

## Competing interests

The authors declare no competing interests with regards to the authorship and/or publication of this article.

## Ethics approval

The authors declare that the patient, whose data is reported in this article, has given written consent to the authors and the Ethics Committee of Krishna Institute of Medical Sciences Deemed University, Karad, India for the publication of this paper.
